# Amyloid degradation mechanisms and potential synergistic effects

**DOI:** 10.4103/NRR.NRR-D-24-01534

**Published:** 2025-03-25

**Authors:** Maksim I. Sulatsky, Olesya V. Stepanenko, Olga V. Stepanenko, Anna I. Sulatskaya

**Affiliations:** Laboratory of Cell Morphology, Institute of Cytology, Russian Academy of Sciences, St. Petersburg, Russia; Laboratory of Structural Dynamics, Stability and Folding of Proteins, Institute of Cytology, Russian Academy of Sciences, St. Petersburg, Russia

Currently, our understanding of the pathogenesis of major neurodegenerative disorders, such as Alzheimer’s, Parkinson’s, and Huntington’s diseases, is largely shaped by the amyloid cascade hypothesis. Particularly, this hypothesis posits that in Alzheimer’s disease, the aggregation of amyloid-beta peptide initiates a series of pathological processes leading to neuronal dysfunction and death (Zhang et al., 2024). Additionally, other mechanistic hypotheses, including tau protein hyperphosphorylation, metal ion dysregulation, and chronic neuroinflammation, contribute to the multifactorial nature of neurodegeneration. Such factors further exacerbate the impact of amyloids accumulation. These protein aggregates represent extremely stable structures that disrupt cellular functioning and initiate cascade inflammatory and oxidative processes, accelerating neurodegeneration. These processes involve microglial activation, inflammatory cytokine release, and impaired synaptic transmission, all exacerbating neuronal damage. Current treatments for neurodegenerative diseases linked to the accumulation of insoluble protein plaques focus mainly on symptomatic relief and slowing disease progression. For example, Alzheimer’s disease treatments often involve cholinesterase inhibitors and NMDA receptor antagonists (Cummings et al., 2023; Zhang et al., 2024). However, these drugs do not address one of the primary causes of degenerative changes—the accumulation of amyloid plaques (Zhang et al., 2023). Efforts to develop drugs targeting the degradation of amyloid fibrils and their aggregates have yet to yield effective and safe treatments. Several new medications following this approach, currently in various stages of development and clinical trials, have demonstrated limited efficacy despite initial optimism. In some cases, these drugs have caused serious side effects, such as brain edema and microhemorrhages (Zhang et al., 2023, 2024; Lasheen et al., 2024; Torres et al., 2024). Thus, despite years of research, no effective treatment has yet been developed to achieve the complete and safe degradation of amyloid fibrils. Challenges in developing an effective therapy for amyloidoses may partly arise from an insufficient understanding of the molecular mechanisms underlying the disruption of pathological aggregates by proposed drugs and the properties of amyloid degradation products. Obtaining such information is crucial for predicting the potential adverse effects of this therapy. In this regard, we aim to analyze the currently known mechanisms of amyloid degradation and evaluate their potential synergistic effects.

**Figure 1:**
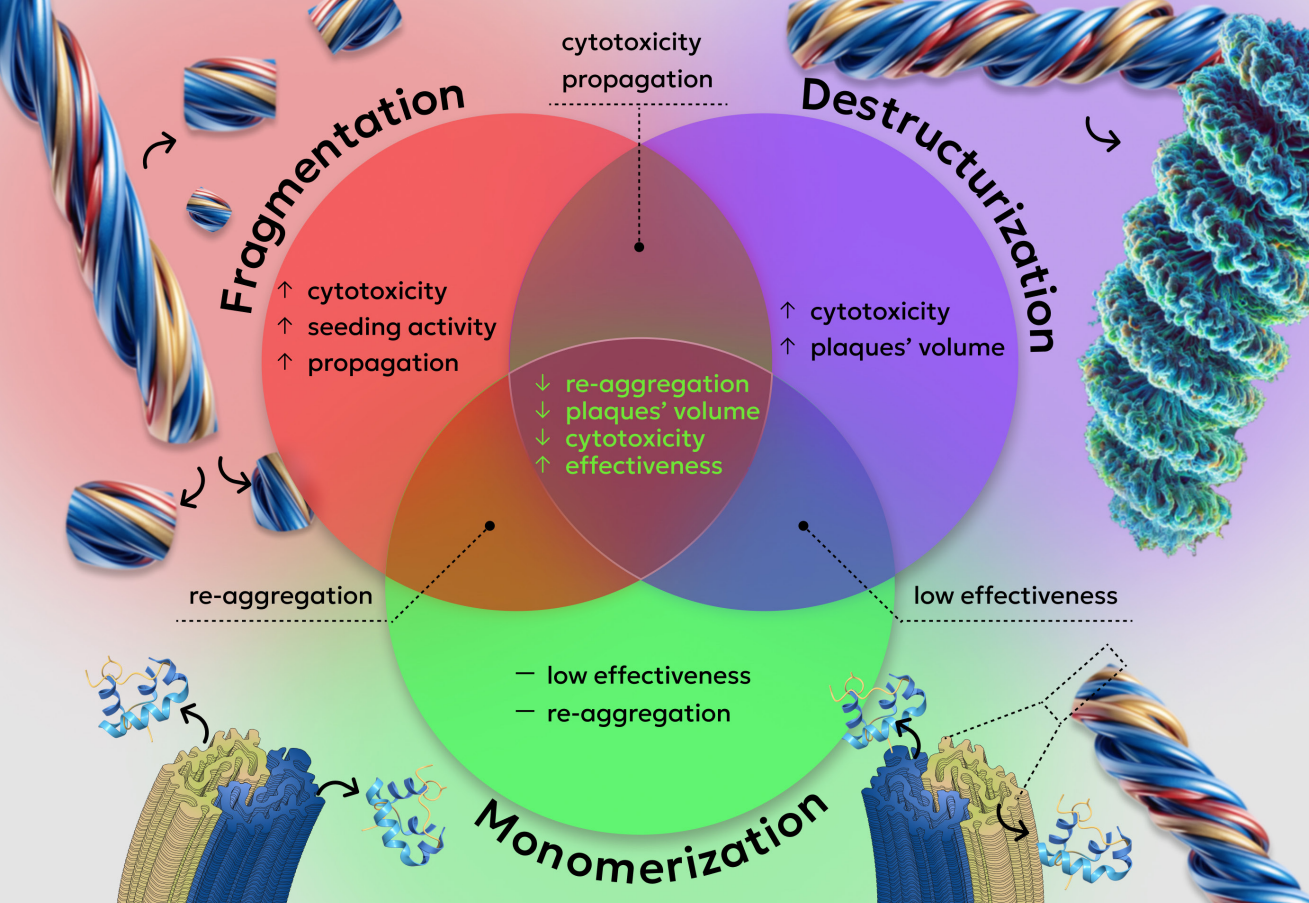
Possible mechanisms of amyloid fibril degradation. Depolymerization of fibrils, the safest method of amyloid degradation, is slow and inefficient, with degradation products retaining their ability to re-aggregate. Destructurization of fibrils without breaking hydrogen bonds in their backbone prevents re-aggregation but increases the volume of fibrillar clusters (plaques) and often increases their cytotoxicity. Fibril fragmentation not only increases cytotoxicity but also enhances seeding activity and accelerates the propagation of amyloids between cells and tissues. Combining these mechanisms in pairs can mitigate or eliminate some of the described side effects. The synergistic combination of all three mechanisms enhances degradation efficiency by reducing aggregate size, preventing re-aggregation, and lowering pathogenicity. Adopting this strategy could greatly enhance the efficacy and safety of newly developed anti-amyloid drugs. Created with Figma and Adobe Photoshop.

**Depolymerization of amyloid fibrils into monomeric subunits:** Disassembling amyloid fibrils into monomeric subunits is regarded as the safest mechanism for degrading these protein aggregates (**Figure 1**). When only this mechanism is implemented, monomers detach from the ends of fibrils, reducing the size of pathogenic structures. This process also increases the number of non-toxic protein species that the body can clear independently. Such a mechanism is likely the primary target for researchers aiming to degrade amyloid aggregates *in vivo*. However, this approach faces significant challenges.

First, a few factors acting exclusively in this manner (without additional effects) have been identified, and these are typically complex, multi-component systems such as chaperone complexes (Kushnirov et al., 2021; Sulatsky et al., 2024). Such chaperone complexes bind aggregated or misfolded proteins by recognizing hydrophobic regions that are typically buried in their native conformations (Kushnirov et al., 2021). This ability allows chaperones to interact with exposed and disordered regions of the proteins at fibril ends, thereby facilitating the release of individual monomeric subunits and promoting partial or complete fibril depolymerization, e.g., via the Hsp70/Hsp40/Hsp110 system in mammalian cells (Sulatsky et al., 2024). Achieving this effect with other systems, particularly single-component ones, remains a highly challenging, if not impossible, task. Second, amyloid fibrils in the body are rarely isolated and they predominantly cluster into dense aggregates. Within such clusters, most of the fibrils’ “free ends” are shielded from the action of large multi-component degrading systems. Thus, even with an effective fibril depolymerization complex, additional loosening of fibrillar clusters is necessary to expose degradation sites on the amyloid. Third, even if the problem of fibrils’ “free ends” accessibility is resolved, their depolymerization remains inefficient due to slow kinetics and the limited number of monomer cleavage sites. Notably, while protein monomers, the amyloid degradation products, are non-toxic to cells, they still pose a potential threat. Detaching from the amyloid fiber and remaining in close proximity to other fibrils (which, as mentioned, are typically part of larger plaques), these monomeric proteins are highly likely to integrate into other fibrils. Even if a method is developed to enhance monomer detachment and prevent re-association, the accumulation of unstructured monomers would likely lead to new toxic structures before being cleared by cellular systems. As a result, the rate of amyloid formation is likely to consistently exceed the rate of their disassembly into monomers.

The described challenges underscore the need for additional agents to complement fibril-depolymerizing factors and achieve a comprehensive solution for complete amyloid degradation: (1) declustering fibrillar aggregates, (2) accelerating monomer detachment, (3) preventing monomer re-aggregation, and (4) enhancing monomer clearance after detachment. In the following sections, we examine potential approaches to overcome these issues.

**Prevention of re-aggregation of fibril-derived monomers and their clearance from the body:** Overexpression of proteolytic enzymes and the use of specific antibodies have been proposed to enhance the clearance of misfolded proteins, including fibril-derived monomers. Proteolysis-targeting chimeras, which leverage the ubiquitin-proteasome system, are among the most promising tools for precise protein targeting and accelerating degradation (Zhang et al., 2024).

Numerous agents have been developed to inhibit fibrillogenesis, including re-aggregation after fibril depolymerization, with proven efficacy *in vivo* and *in vitro*. These agents, including small molecules of various origins and peptide inhibitors, bind to monomers and prevent further interactions (Kumar et al., 2012; Zhang et al., 2023, 2024). These molecules act as “traps” for monomers, stabilizing them and preventing re-aggregation. However, despite their targeted delivery, such inhibitors often suffer from poor pharmacokinetics, solubility, and stability. Efforts to address this issue include coupling inhibitory agents with nanoliposomes, which can improve their properties.

Thus, strategies developed earlier to prevent aggregation can also be applied to prevent re-aggregation and facilitate the clearance of fibril-derived monomers. However, these approaches will only be effective if key challenges, such as facilitating amyloid monomerization by declustering fibrillar aggregates and increasing fibril monomerization rate are adequately addressed.

**Destructurization of individual amyloid fibers and loosening fibrillar clusters:** One approach to declustering dense amyloid aggregates is the so-called destructurization of fibrils (**Figure 1**). This process is achieved by disrupting various intramolecular interactions (hydrophobic, ionic, and covalent). Notably, the hydrogen bonding network in the fibril core maintains its integrity and even can experience enhanced stabilization (Sulatsky et al., 2024). As a result, the amyloid fibers do not shorten but become less compact and occupy a larger volume (essentially “fluffing”), which contributes to the “loosening” of fibrillar clusters and, consequently, increases the accessibility of sites for other agents with distinct mechanisms of action. Specifically, this process improves the availability of fibril “free ends,” enhancing their depolymerization efficiency by chaperone complexes. Different agents achieve fibril “fluffing” through distinct mechanisms. Some small molecules bind specifically to fibrils, inducing conformational changes that weaken internal fibril interactions. Others act on the fibril surface, reducing fibril compactness and promoting structural loosening (Stepanenko et al., 2020).

However, destructurization of fibrils alone cannot fully degrade or reduce the cytotoxicity of amyloids. On the contrary, in some cases, this mechanism of degradation has been shown to increase their pathogenicity. For instance, this was demonstrated in the case of the small heat shock protein alpha-B-crystallin (Stepanenko et al., 2020). The increased cytotoxicity may be attributed to several factors. One aspect of amyloid toxicity is disrupting membrane integrity through strong associations with membranes, which reduces cell proliferation and migration rates (Milanesi et al., 2012). Additionally, amyloids interact with membrane receptors, triggering signaling cascades that cause cell damage or death. Morphological changes during fibril destructurization may increase amyloid membrane affinity by exposing or shielding specific protein regions. Larger loosened aggregates interact with greater cell surface areas, causing more damage and disrupting cellular functions. Partial proteolytic cleavage of unfolded protein fragments can mitigate the adverse effects of loosening amyloid clusters. This approach can neutralize their contribution to increased cytotoxicity. Such a “dual” mechanism has recently been demonstrated during the action of extracellular matrix metalloproteinase-9 on fibrils (Stepanenko et al., 2024).

Thus, destructurization of fibrils without increasing their cytotoxic effect can play a key role in preparing fibrils for further degradation by making clusters less compact and amyloid fibers more accessible to other degrading agents.

**Fragmentation of amyloids:** Increasing the number of fibrils “free ends” is another approach to accelerating fibril depolymerization. This can be achieved through the fragmentation of amyloid fibrils, i.e., cutting large aggregates into smaller fragments (**Figure 1**). Fragmentation can be achieved by various means, including mechanical treatments (e.g., ultrasound) or partial proteolytic cleavage (e.g., by trypsin; Stepanenko et al., 2021; Sulatsky et al., 2024). These effects by different mechanisms ultimately lead to the breaking of hydrogen bonds between beta-strands in the fibril backbone and the cutting of longer fibrils into shorter segments. Fibril fragmentation can enhance the efficiency of complexes that disassemble amyloids into monomers by several orders of magnitude.

However, fragmentation also has its own “side effects.” This process typically increases seeding activity and cytotoxicity, as more fibril fragments create additional “amyloid matrices,” accelerating aggregation. Additionally, it facilitates the propagation of “amyloid hotspots” between cells and tissues. Research further indicates that short fibril fragments more effectively disrupt membrane integrity and trigger inflammatory reactions than larger amyloid aggregates (Sulatsky et al., 2024). Short fragments readily penetrate cells, exacerbating adverse effects. Specifically, they can cause an increase in reactive oxygen species, resulting in oxidative stress and further cellular damage. Thus, fragmentation alone without supplementary mechanisms may be counterproductive and could exacerbate pathological effects. A potential solution is combining fibril-fragmenting agents with other factors that dismantle their structure, inhibit their seeding potential, and enlarge them to prevent cellular penetration.

**A step forward - combined therapy leveraging synergistic amyloid degradation mechanisms:** our analysis revealed that amyloid degradation mechanisms fall into three main categories: destructurization, fragmentation, and monomerization (depolymerization into monomeric subunits; **Figure 1**). Individually, each of these mechanisms has inherent limitations and potential side effects. For instance, incomplete amyloid degradation often produces fibril fragments or loosened clusters of aggregates that are more pathogenic than intact amyloids. This significantly restricts the safety and efficacy of current drug development efforts. A promising solution to these challenges is a combined approach to complete amyloid fibril degradation. This strategy aims to overcome the limitations of existing methods and create a new generation of drugs for treating various forms of amyloidosis, including those associated with neurodegenerative diseases.

Our proposed strategy combines all three mechanisms, ensuring control over degradation product properties, their stabilization, and rapid clearance from the body (**Figure 1**). Specifically, the destructurization of amyloids enhances degradation site accessibility for fibril-fragmenting enzymes, fragmentation accelerates depolymerization, and stabilization with rapid monomer removal prevents re-aggregation. This combined approach leverages the synergistic effect of different mechanisms, significantly enhancing therapeutic efficiency compared to individual methods, while minimizing limitations and side effects. In our view, this multi-layered strategy, capable of achieving more efficient and safer amyloid degradation, represents a critical step forward in improving outcomes for patients. However, a combined strategy requires careful monitoring at every stage, including cytotoxicity evaluation, inflammatory response control, and oxidative stress assessment. Such comprehensive monitoring will ensure maximum therapeutic safety and minimize patient risk.

**Perspective on combined therapy for amyloidoses treatment:** This study proposes a combined strategy for destroying pathological protein aggregates, based on an analysis of accumulated data on amyloid degradation mechanisms, their advantages, and limitations. Unlike existing methods, this strategy focuses on completely breaking down amyloids and preventing toxic intermediate products, making it both more effective and safer. One of the key elements of the proposed strategy is its adaptability to individual patients. Personalized medicine is a crucial direction in modern therapy, and this combined approach can be tailored to the disease stage, patient characteristics, and pathology severity, including amyloid accumulation levels. This adaptability maximizes the advantages of each degradation mechanism while minimizing side effects and enhancing treatment safety. A personalized, multi-level approach to amyloidoses therapy could radically improve patient outcomes, enhancing quality of life and offering hope for recovery. For the successful implementation of combined therapies against amyloidoses, further in vitro investigations are essential. These studies should focus on identifying and developing new anti-amyloid agents, clarifying how known amyloid-degrading substances act, determining their optimal working concentrations and conditions for high efficacy, and establishing the most advantageous combinations and sequence of degradation stages (including both simultaneous and sequential application of agents). Such efforts will pave the way for significant progress in addressing the problem of systemic and localized amyloidoses, including those associated with neurodegenerative diseases.

*The work was funded by the Russian Science Foundation (grant No. 23-74-10092) (to AIS).*

**Additional file:** *Open peer review reports 1 and 2.*

OPEN PEER REVIEW REPORT 1

OPEN PEER REVIEW REPORT 2
