# Supplementary material for: Amyloid degradation mechanisms and potential synergistic effects
Source: Neural Regen Res. 2025 Mar 25;21(5):1981–2. doi: 10.4103/NRR.NRR-D-24-01534 (PMC12694633; doi:10.4103/NRR.NRR-D-24-01534)
Supplement: Supplementary file 1 [file NRR-21-1981_Suppl1.pdf]

OPEN PEER REVIEW REPORT 1

Name of journal: Neural Regeneration Research
Manuscript NO: NRR-D-24-01534
Title: Divided fall, united stand: a synergistic strategy for amyloid fibril degradation
Reviewer's Name: Christopher V. Synatschke
Reviewer's country: GERMANY

COMMENTS TO AUTHORS

The authors nicely summarize the underlying problem of how to remove amyloid plaques and the challenges that are associated with achieving the removal of amyloid plaques in vitro, or even in a patient.

The only minor comment that I have to improve the manuscript further is that some sections could benefit from a brief explanation on how the mechanism of action of a particular approach works. For example:

- How is a fragmentation of amyloids achieved? Is it mechanical force, disruption of hydrogen bonds, or something else?
- What are the reasons that chaperones can depolymerize fibrils? Is the binding affinity of amyloids higher towards the chaperone than towards other fibrils?
- How does a destructurization work and is it the same for most drugs that belong to this class, or are different mechanisms at play?
