# Supplementary material for: Amyloid degradation mechanisms and potential synergistic effects
Source: Neural Regen Res. 2025 Mar 25;21(5):1981–2. doi: 10.4103/NRR.NRR-D-24-01534 (PMC12694633; doi:10.4103/NRR.NRR-D-24-01534)
Supplement: Supplementary file 2 [file NRR-21-1981_Suppl2.pdf]

OPEN PEER REVIEW REPORT 2

Name of journal: Neural Regeneration Research
Manuscript NO: NRR-D-24-01534
Title: Divided fall, united stand: a synergistic strategy for amyloid fibril degradation
Reviewer's Name: Gefei Chen
Reviewer's country: SWEDEN

COMMENTS TO AUTHORS

The manuscript presents analysis of current strategies for the degradation of amyloid fibrils, focusing on the challenges and potential of various methods in treating amyloidosis associated with neurodegenerative disorders. It is well-written and concise, providing a clear overview of a complex topic. However, there are several areas where the manuscript could be further strengthened:

1) The manuscript should begin with a clear and detailed explanation of the amyloid cascade hypothesis, alongside other mechanistic hypothesis of neurodegenerative diseases. This would provide a comprehensive backdrop, setting the stage for discussing the relevance and impact of amyloid fibril degradation strategies in a broader context neurodegenerative disorders.

2) While the manuscript proposes an combined therapy leveraging synergistic amyloid degradation mechanisms, it lacks detailed discussion on how these strategies could be practically implemented. For instance, it would be beneficial to describe specific methods for combining different compounds or agents. Clarifying whether these strategies involve simultaneous or sequential application of therapies could also be valuable.
